# TECHNOLOGY DESIGN TO SUPPORT SOCIAL ENGAGEMENT FOR OLDER ADULTS

**DOI:** 10.1093/geroni/igz038.769

**Published:** 2019-11-08

**Authors:** Wendy Rogers

**Affiliations:** University of Illinois, Urbana-Champaign, Champaign, Illinois, United States

## Abstract

Evidence that loneliness and isolation are precursors to myriad negative health issues is accumulating. For older adults, social engagement may be particularly important due to life-changing events that can accompany older adulthood, including retirement, disease, or mobility limitations. Individuals vary in their levels of social engagement, and these differences may have consequential effects on quality of life. Technology has the potential to create and enhance social engagement opportunities for older adults at risk for social isolation. For such technology innovations to be effective and widely adopted, designers must consider the unique needs, capabilities, limitations, and preferences of older adults. I will describe technologies that currently exist (e.g., apps, mobile devices, social networking) or are being developed (e.g., robotics, telepresence, virtual reality) to support social engagement, connectedness, and community participation. I will discuss the potential of these technologies as well as the design and training challenges unique to older adults.

